# Precipitation predictability affects intra- and trans-generational plasticity and causes differential selection on root traits of *Papaver rhoeas*

**DOI:** 10.3389/fpls.2022.998169

**Published:** 2022-11-14

**Authors:** Martí March-Salas, J. F. Scheepens, Mark van Kleunen, Patrick S. Fitze

**Affiliations:** ^1^ Plant Evolutionary Ecology, Faculty of Biological Sciences, Goethe University Frankfurt, Frankfurt am Main, Germany; ^2^ Department of Biodiversity and Evolutionary Biology, Museo Nacional de Ciencias Naturales (MNCN-CSIC), Madrid, Spain; ^3^ Department of Biodiversity and Ecologic Restoration, Instituto Pirenaico de Ecología (IPE-CSIC), Jaca, Spain; ^4^ Ecology, Department of Biology, University of Konstanz, Konstanz, Germany; ^5^ Zhejiang Provincial Key Laboratory of Plant Evolutionary Ecology and Conservation, Taizhou University, Taizhou, China

**Keywords:** acquisition strategy, adaptive transgenerational plasticity, annual plants, inter-seasonal predictability, multi-generation experiment, root allocation strategy, root functional traits, selection gradients

## Abstract

Climate forecasts show that in many regions the temporal distribution of precipitation events will become less predictable. Root traits may play key roles in dealing with changes in precipitation predictability, but their functional plastic responses, including transgenerational processes, are scarcely known. We investigated root trait plasticity of *Papaver rhoeas* with respect to higher *versus* lower intra-seasonal and inter-seasonal precipitation predictability (*i.e.*, the degree of temporal autocorrelation among precipitation events) during a four-year outdoor multi-generation experiment. We first tested how the simulated predictability regimes affected intra-generational plasticity of root traits and allocation strategies of the ancestors, and investigated the selective forces acting on them. Second, we exposed three descendant generations to the same predictability regime experienced by their mothers or to a different one. We then investigated whether high inter-generational predictability causes root trait differentiation, whether transgenerational root plasticity existed and whether it was affected by the different predictability treatments. We found that the number of secondary roots, root biomass and root allocation strategies of ancestors were affected by changes in precipitation predictability, in line with intra-generational plasticity. Lower predictability induced a root response, possibly reflecting a fast-acquisitive strategy that increases water absorbance from shallow soil layers. Ancestors’ root traits were generally under selection, and the predictability treatments did neither affect the strength nor the direction of selection. Transgenerational effects were detected in root biomass and root weight ratio (RWR). In presence of lower predictability, descendants significantly reduced RWR compared to ancestors, leading to an increase in performance. This points to a change in root allocation in order to maintain or increase the descendants’ fitness. Moreover, transgenerational plasticity existed in maximum rooting depth and root biomass, and the less predictable treatment promoted the lowest coefficient of variation among descendants’ treatments in five out of six root traits. This shows that the level of maternal predictability determines the variation in the descendants’ responses, and suggests that lower phenotypic plasticity evolves in less predictable environments. Overall, our findings show that roots are functional plastic traits that rapidly respond to differences in precipitation predictability, and that the plasticity and adaptation of root traits may crucially determine how climate change will affect plants.

## Introduction

Current climate change entails rising global temperatures, longer and more frequent dry periods, and changes in weather predictability (Xu et al., 2020; IPCC, 2021). Although global mean precipitation is expected to remain rather constant in the near future, precipitation patterns will change, leading to a reduction in precipitation predictability at different temporal scales, *i.e.*, among days, weeks, seasons or years (Tonkin et al., 2017; Xu et al., 2020). The degree of intrinsic precipitation predictability (*i.e.*, the degree of temporal autocorrelation among precipitation events; Pennekamp et al., 2019) will determine the amount and timing of water availability for plants. This may cause plants to change their nutrient and water acquisition strategy, plastically modulate their traits, and eventually may result in adaptation to the new conditions (Yin et al., 2022).

According to evolutionary theory, the evolution of increased plasticity would be favoured under temporally variable but predictable environments (Caro et al., 2016; McNamara et al., 2016). In contrast, reduced plasticity would evolve in temporally variable but less predictable environments, since plastic changes may be misaligned with differential selective pressures over generations (Lande, 2009; Botero et al., 2015; Tufto, 2015; Leung et al., 2020). A recent experiment suggested that higher predictability in natural environments may contribute to the evolution of transgenerational plasticity in reproductive traits (*i.e.*, to the offspring’s plastic response to the ancestor’s environment) (Yin et al., 2022). Other experiments showed that transgenerational responses in phenological and fitness-related traits to different degrees of predictability are possible (Franch-Gras et al., 2017; March-Salas and Fitze, 2019; March-Salas et al., 2019; Colicchio and Herman, 2020; March-Salas et al., 2021a).

Evolutionary ecology has primarily focused on phenological (e.g. flowering start) and performance (e.g. aboveground biomass, number of flowers, number of seeds) traits to determine how plants adapt to environmental changes. However, root traits may also play an important role for overall plant development and tolerance of or resistance to changing environmental conditions. Root traits could be under strong natural selective pressure, and vary over generations reflecting adaptation to dynamic water availability (Zhou et al., 2019; Yamauchi et al., 2021). Roots can also rapidly sense fluctuations in water availability and may help species to deal with temporal shifts in the means and predictability of precipitation (Zhou et al., 2019; March-Salas et al., 2021b). However, how the maternal environment determines the transgenerational expression of the root traits of progeny and adaptive responses in future environments remains largely unknown (Donelson et al., 2018).

Plastic responses of roots can however be costly (DeWitt et al., 1998), for instance through re-allocation of resources from other plant structures to roots (Manenti et al., 2015). A reduction of resource investment into root biomass may be a response to stressful conditions, potentially helping plants to maintain or even increase their short-term performance by re-allocation into above-ground structures (Lundgren and Des Marais, 2020). Additionally, in response to specific environmental cues, plants may promote within-root allocation-shifts. For instance, a constant water availability may favor deeper and bigger roots and increased branching density but fewer fine roots (Wu et al., 2016; March-Salas et al., 2021b). Promoting lateral root branching while reducing primary root depth may also help plants to withstand stressful conditions such as water limitation or high salinity levels (Ambastha et al., 2020; Gallego-Tévar et al., 2022). This should be especially true in fast-cycling plants with acquisitive strategies, since their roots should acquire temporally available resources in shallow soil layers to guarantee a rapid increase in performance (Hermans et al., 2006; Weemstra et al., 2016; Gallego-Tévar et al., 2022). Despite their relatively small root systems, many annual plants use acquisitive strategies to favour rapid growth and fast reproduction under stressful conditions (Gallego-Tévar et al., 2022). So, evolutionary experiments that focus on root traits are needed to increase our understanding of how plants deal with changing environments.

**Figure 1:**
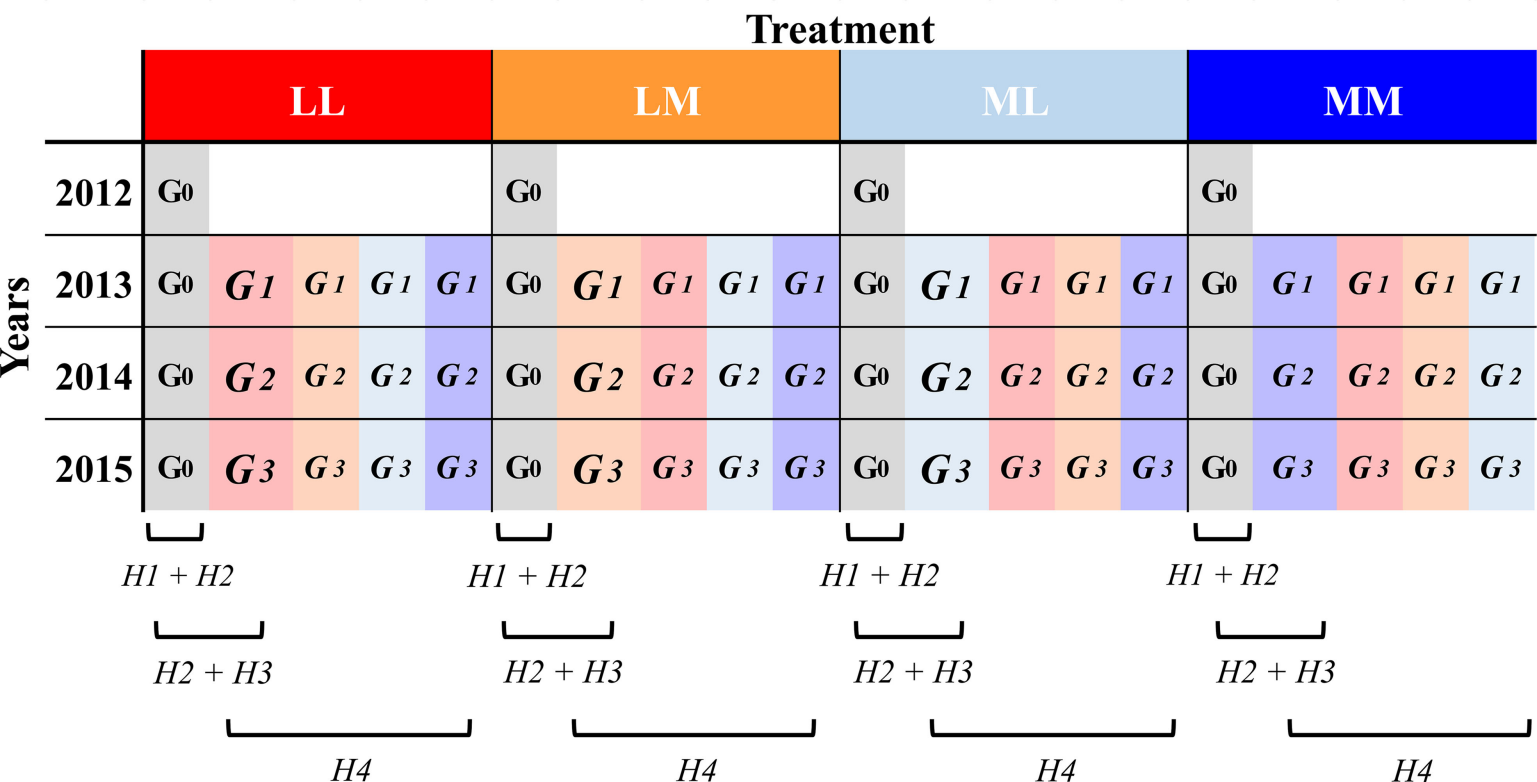
Design of the evolution experiment. Ancestors (G_0_) and the three descendant generations (G_1-3_) were subjected to four different precipitation predictability treatment combinations (LL, LM, ML, MM; *see* methods) in their year of growth (*i.e.*, Treatment). Ancestors (in grey) were grown from 2012 to 2015 and descendants (highlighted in the maternal treatment colour but lighter than the treatment in the growing year) from 2013 to 2015. Below, the brackets refer to the experimental lines (*i.e.*, generation and treatment) involved in the assessment of each of the four main hypothesis (*H*) we addressed in this work (*see* Introduction).

In a multi-generation experiment with the annual herb *Papaver rhoeas* L. (Papaveraceae) grown under semi-natural outdoor conditions, we manipulated intra-seasonal (among days) and inter-seasonal (among spring and summer) precipitation predictability. We grew four consecutive generations, each year in the same as well as in contrasting predictability regime to which the maternal plants were exposed. In addition, the ancestral generation (the original generation that had not experienced any of the experimental treatments) was sown in each of the four years under all predictability regimes. With this experimental design, first, we investigated whether and how roots of ancestors respond plastically (*i.e.*, intra-generational plasticity; e.g. Sobral et al., 2021) to more *versus* less intra- and inter-seasonal precipitation predictability within four consecutive experimental years. Second, we tested whether root traits are subject to selection. Third, we tested for transgenerational effects to the different predictability regimes (*i.e.*, genetically as well as non-genetically inherited effects when plants grow in the same treatment over generations; March-Salas et al., 2019). In other words, by comparing ancestors and descendants in the same year and treatment, we investigated whether precipitation predictability promoted trait differentiation in descendants, and whether there was a change in the root trait response between ancestors and descendants that is mediated by the precipitation predictability treatment. This would point to a transgenerational response to a specific environment. Here, we also tested whether the observed differences in root traits between ancestors and descendants led to higher performance of the descendants, what would point to an adaptive change. Fourth, by comparing descendants that grew in the same or contrasting treatment as their mother, we tested whether the descendants’ response to the treatments depended on the maternal treatment, which will point to differences in transgenerational plasticity due to the predictability regime (**Figure 1**).

We hypothesized (H1) that roots of ancestors plastically respond to different degrees of precipitation predictability, with greater root response and investment in root biomass in environments that fluctuate less predictably in order to make instantaneous use of temporally rare events of water availability; (H2) that the considered root traits (maximum rooting depth, number of secondary roots, root biomass, and the relative investment to each of them) affect plant fitness and are under strong selection; (H3) that differences between ancestors and descendants in root traits occur when inter-generational predictability is high and when current environmental conditions are more predictable; and (H4) that transgenerational plasticity is favoured in more predictable conditions.

## Material and methods

### Experimental setup

This study was conducted during four consecutive years (2012-2015) under natural conditions at the experimental field station ‘El Boalar’ (42°33′N, 0°37′W, 705 m a.s.l.; Jaca, Huesca, Spain). The experimental setup consisted of 16 enclosures of 100 m^2^ each, which were covered and separated with a mesh and a 1 m tall metal wall. The setup included an automated watering system consisting of one sprinkler in each of the four corners of each enclosure, providing homogenous precipitation in the whole enclosure and allowing us to control the quantity and timing of the watering events (March-Salas et al., 2019). Within each of these enclosures, we established a plot of 7.2 m^2^ that was surrounded by a vertical mosquito net (30 cm aboveground and 10cm belowground) to protect plants from herbivory (e.g. snails, slugs). In each experimental year, the top 30 cm of the soil of each plot was manually ploughed, loosened, homogenised and flattened before sowing.

### Study species

We used the annual herb *Papaver rhoeas* L. (common poppy Papaveraceae), a wide-spread and fast-growing species with acquisitive and exploitative strategies (McNaughton and Harper, 1964; Pérez-Ramos et al., 2019), which naturally occurs in the surroundings of the study site. The species’ height is between 10 and 50 cm, its flowers are bright red and its fruits are a capsule that usually contain hundreds of >1 mm long seeds (Franklin-Tong and Franklin, 1992). It has a slender primary root with multiple lateral secondary roots (**Figure S1**). The original seed lot of *P. rhoeas* was obtained in 2011 from 50 maternal plants of a nearby population (~60 km away) that experiences higher humidity and otherwise a similar climate as the study site (March-Salas et al., 2019). The seeds from the original seed lot were mixed and are hereafter referred to as ancestors or the ancestral generation. Ancestral seeds were sown in each of the treatments in the four experimental years to be able to disentangle treatment-differences among years from overall treatment effects as well as year control (*see* below). To preclude potential maternal bias in genetic differentiation among ancestors, we accounted for differences in average seed weight and in the coefficient of variation (CV) in seed weight among the groups of seeds that we sowed in the different treatments and years, *i.e.*, we grouped the seeds so that there were no differences in seed weight mean and CV between the 16 groups – 4 treatment combinations × 4 years.

### Precipitation predictability treatment

The field site’s natural precipitation predictability was manipulated by means of the automated watering system, to simulate higher *versus* lower intrinsic precipitation predictability at two different temporal scales: intra-seasonal (among days) and inter-seasonal (between spring and summer) predictability.

More (M) and less (L) predictable intra-seasonal predictability was simulated by applying 14 supplemental watering events each week at regular or random time-intervals, respectively (March-Salas et al., 2019). Both treatments consisted of the same total amount of precipitation (natural + supplemental) but exhibited a different degree of temporal autocorrelation among precipitation events, *i.e.*, they contrasted in their intrinsic precipitation predictability. Differences in intrinsic predictability of precipitation was calculated by means of permutation entropy, which is inversely related to intrinsic predictability (Pennekamp et al., 2019). The combination of natural and experimental precipitation resulted in a permutation entropy of 0.86 in L and 0.77 in M. Permutation entropy in M was equal to the one of natural precipitation (March-Salas et al., 2019). L thus had 11.69% lower intrinsic precipitation predictability than M (and than natural precipitation). The simulated subtle but significant differences in predictability are in line with theoretical conditions that should lead to adaptive transgenerational plasticity: existence of natural environmental heterogeneity and predictability of an intermediate frequency and without extreme events (Burgess and Marshall, 2014; Uller et al., 2015; Yin et al., 2022).

In spring of each year, from April to late June (hereafter referred to as ‘early treatment’), eight plots were subjected to M and another eight plots to L predictable predictability. In summer, from July to late September (hereafter referred to as ‘late treatment’), half of the plots of each early treatment were exposed to the same (MM, LL) or to the other (ML, LM) treatment, simulating higher (MM, LL) and lower (LM, ML) inter-seasonal predictability. This thus resulted in a two-factorial design with four precipitation predictability treatment combinations: (1) more predictable precipitation during the whole experimental period (MM), (2) less predictable during the whole experimental period (LL), (3) more predictable during spring and less predictable during summer (ML), and (4) less predictable during spring and more predictable during summer (LM). Each treatment combination was applied to four independent plots.

In each experimental year, the total precipitation (natural + supplemental) falling in the experimental set up was within the natural range recorded: (a) at the field site in the previous ten years, (b) in the area of origin of the ancestor seeds, and (c) in the species distribution range (McNaughton and Harper, 1964; March-Salas et al., 2019; March-Salas et al., 2021a). The study site had high potential evapotranspiration and plants were exposed to water stress from April to September (**Figure S2**). In the experimental plots, plants were as well exposed to water stress, although less pronounced (for further information on climatic and soil conditions of the study site, *see* supplementary text and **Figure S2**).

### Sowing procedure and evolution experiment

In each of the four experimental years, seeds of *P. rhoeas* were individually sown in April in each of the 16 plots. In each plot, three seeds were planted in each of 84 planting positions aligned in a quadratic grid and separated by 20 cm. Each position was differentiated with a metal stick, which was labelled with the seed origin. When more than one seed emerged per position, seedlings were thinned to one.

In 2012, randomly selected ancestor seeds were planted in the 84 positions, and from 2013 to 2015, ancestor and descendant seeds (*i.e.*, the descendant seed generation 1 in 2013, generation 2 in 2014 and generation 3 in 2015) were both sown in the same plot (March-Salas et al., 2019). More specifically, from 2013 to 2015, randomly selected ancestor seeds were sown in 21 positions per plot and descendant seeds in 63 positions per plot. In the case of descendants, from each maternal line (*i.e.*, descendants originating from the same mother) seeds were sown in the same treatment combination as their mother and also in the other three treatment combinations. In the first descendant generation, we included seeds from 12 maternal plants per treatment line, except for seeds from the ML treatment, for which only seeds from 3 maternal plants could be included given that all other maternal plants did not produce enough seeds. For the subsequent generations, we included seeds from 6 maternal plants per treatment line. To avoid bias among used and not used mothers and among used and not used seeds of the same mother, we tested that there were no phenotypic differences between selected and not selected mothers, no differences in seed weight between selected and not selected seed lots, and no significant interaction between the factor ‘selected/not selected’ and the treatments combinations (all *p* ≥ 0.2). Moreover, to make sure that treatment-induced transgenerational effects are not confounded with transgenerational responses to plot-specific conditions, seeds were never planted in the plot in which their mother had been growing previously.

### Plant measurements

At the end of a plant’s life cycle (*i.e.*, when all fruits of a given plant were ripe), we individually harvested above-ground biomass and root biomass. Roots were carefully dug out and washed to remove the substrate (**Figure S1**). For each plant, the length of the longest root was measured as an estimate of maximum rooting depth (precision: 1 mm) and the number of secondary roots (all lateral root branches >0.5 cm long) was counted. The dry mass of the above-ground and root biomass (in g) was determined after drying at 50 °C for three days. To test whether the treatments affected root allocation strategies, the following root traits were calculated: (1) root weight ratio (RWR; root biomass/total biomass); (2) relative rooting depth (maximum rooting depth/root biomass); and (3) relative root branching (number of secondary roots/root biomass).

### Statistical analysis

Four main types of analysis were conducted using *R version 4.0.3* (R Development Core Team, 2020) and Linear Mixed-effects Models (LMMs) implemented in the *lme4* package and the *lmer* function (Bates et al., 2015).

First (H1), we tested for treatment effects on root traits of the ancestral generation across the four years (*i.e.*, intra-generation plasticity across four years). The six measured traits (root biomass, maximum rooting depth, number of secondary roots, RWR, relative rooting depth and relative root branching) were included as response variables in six separate models. As fixed factors, we included early treatment (less predictable *versus* more predictable), late treatment (less predictable *versus* more predictable), year (2012, 2013, 2014, 2015) and their two- and three-way interactions, and as random factor we included ‘plot_year’ (a factor referring to the plot × year combination), as described in Bates et al. (2015). A significant treatment effect would indicate intra-generational plasticity; a significant year effect would indicate differences among years irrespective of the treatment, and a significant treatment(s) × year interaction would indicate that the treatment effect depends on the year.

Second (H2), the selective regime (the type and the strength of natural selection) to which the root traits were exposed was analysed using selection gradients (Lande and Arnold, 1983). Analysing the selective regime allows to test if the measured root traits affect plant performance, and therefore, whether they can be considered as functional traits (Violle et al., 2007). As a performance proxy, we used total biomass, because it is generally positively related to seed number (Shipley et al., 2016; Younginger et al., 2017). We could not have precise data on seed number for all plants due to potential loss of seeds from capsules that had opened before collection. However, for the collected data, seed number was strongly correlated with total plant biomass (*F*_1,455_ = 249.48, *P* < 0.001, *ρ* = 0.6). Prior to the analyses, total biomass and all root variables were *ln*-transformed and standardized to a mean of zero and a standard deviation of one to obtain standardized selection gradients (Lande and Arnold, 1983). First, only ancestral plants were analysed. Early treatment, late treatment and year (2012, 2013, 2014, 2015) were modelled as fixed factors, and the root trait of interest was included as a covariate. To account for non-independence of plants measured in the same year and in the same plot, ‘plot_year’ was included as random factor. To test for directional, stabilizing or disruptive selection, linear and quadratic terms of the root variable of interest were included. The full model also contained all two- and three-way interactions between treatments and the covariates. To test whether selective regimes differed among ancestors and descendants, an additional set of analyses was conducted in which ancestors and descendants both sown in 2013, 2014 and 2015 were analysed together. In these analyses, the generation (ancestor *versus* descendant) and its interactions with the other variables were added to the above-mentioned model as well as the ID of the maternal line as random effect.

Third (H3), we tested whether root traits of the descendant generations differed from those of the ancestral generation grown in the same year, and whether differences depended on the predictability treatment. We included early treatment, late treatment, generation (ancestral *versus* descendants), year (2013, 2014, 2015) and their two-, three-and four-way interactions as fixed factors. The ID of the maternal line and ‘plot_year’ were modelled as random factors. A significant generation factor would indicate differences between ancestors and descendants independent of treatment, and a significant generation × treatment interaction would indicate that predictability treatment effect depends on the studied generation.

Fourth (H4), we tested whether treatment effects in descendants depended on their mothers’ treatment by only analysing descendants. The maternal treatment combination, the descendants’ treatment combination, the two-way interaction, and year were included as fixed factors, whereas the ID of the maternal line and ‘plot_year’ were included as random factors. A significant maternal treatment effect would indicate that the maternal treatment manifested in the descendants independent of the descendant treatments; a significant descendant treatment effect would indicate treatment differences independent from maternal environment; a significant maternal × descendant treatment interaction would indicate that the outcome of the descendants’ treatment depends on the treatment to which their mothers were exposed, pointing to transgenerational plasticity. Moreover, we calculated the coefficient of variation (the ratio of the standard deviation to the mean, based on means, CV_m_) among treatments in descendants for each maternal treatment as a quantitative estimator of plasticity (Schlichting, 1986; Valladares et al., 2000), as well as the overall CV of ancestors and of descendants (**Table S1**).

In all models, we tested the assumptions of normality and homogeneity of variance of the residuals using the Shapiro-Wilk test and the Bartlett test, respectively. If the residuals were not normally distributed, we transformed the response variable (see **Tables 1**, **2**). In the case of heteroscedasticity, we applied a weighted least square regression (Strutz, 2016) by including weights (1/variance) into the model, using the extract model *weights* command. Whenever there were significant main effects containing more than two factor levels or significant interactions, we applied Tukey’s *post-hoc* contrasts using the *lsmeans* package to understand which levels differed (Lenth, 2016). Moreover, in all statistical analyses, the most parsimonious model was determined using stepwise backward elimination. Sample size per treatment, year and generation of ancestors, pure descendant lines, and descendants from all treatment combinations are shown in **Table S2**.

**Table 1:** Precipitation predictability treatment effects on root traits of ancestors.

		N of secondary roots			Maximum rooting depth ^a^			Root biomass ^b^		
Parameter	df	Chi^2^	P		Chi^2^	P		Chi^2^	P	
Early [M]	1	0.693	0.405		0.931	0.335		1.311	0.252	
Late [M]	1	1.669	0.196		0.000	0.995		1.736	0.188	
Year	3	140.493	<0.001	***	109.640	<0.001	***	76.550	<0.001	***
Early × Late	1	4.825	0.028	*	1.821	0.177		2.805	0.094	.
Early × Year	3	2.697	0.441		2.063	0.559		4.659	0.199	
Late × Year	3	9.121	0.028	*	6.172	0.104		13.488	0.004	**
Early × Late × Year	3	1.115	0.773		2.980	0.395		16.777	<0.001	***
		**Root weight ratio ^c^**			**Relative root branching ^d^**			**Relative rooting depth ^d^**		
**Parameter**	**df**	**Chi^2^**	**P**		**Chi^2^**	**P**		**Chi^2^**	**P**	
Early [M]	1	0.484	0.487		8.647	0.003	**	1.140	0.286	
Late [M]	1	0.437	0.509		1.717	0.190		10.517	0.001	**
Year	3	27.642	<0.001	***	13.595	0.004	**	29.809	<0.001	***
Early × Late	1	1.610	0.205		0.233	0.630		1.563	0.211	
Early × Year	3	4.091	0.252		3.008	0.390		4.478	0.214	
Late × Year	3	2.972	0.396		3.081	0.299		7.102	0.069	.
Early × Late × Year	3	55.815	<0.001	***	3.319	0.174		6.285	0.094	.

transformations: ^a^^0.4; ^b^^0.1; ^c^^0.6; ^d^log. Linear Mixed-effects Models (LMMs) included Early treatment, Late treatment, Year and their two- and three-way interactions. Transformations applied to the response variable are indicated. Significance is shown as * 0.05 > *P* ≥ 0.01; ** 0.01 > *P* ≥ 0.001; *** *P <* 0.001, and . reflects marginal effects (0.1 > *P* ≥ 0.05). Sample size was 458. Response variables are number (*N*) of secondary roots, maximum rooting depth, root biomass, root weight ratio (RWR), relative root branching, and relative rooting depth.

**Table 2:** Root transgenerational plasticity to precipitation predictability treatments.

		N of secondary roots ^a^			Maximum rooting depth ^b^			Root biomass ^c^		
Parameter	df	Chi^2^	P		Chi^2^	P-value		Chi^2^	P	
Year	2	162.112	<0.001	***	2488.959	<0.001	***	85.861	<0.001	***
Maternal treatment	3	3.925	0.270		5.762	0.124		2.612	0.455	
Descendants treatment	3	6.636	0.084	.	6.460	0.091	.	5.853	0.119	
Maternal × Descendants	9	10.625	0.302		17.715	0.039	*	20.247	0.016	*
		**Root weight ratio *^b^***			**Relative root branching *^a^***			**Relative rooting depth *^a^***		
**Parameter**	**df**	**Chi^2^**	***P***		**Chi^2^**	***P***		**Chi^2^**	***P***	
Year	2	5.217	0.074	.	16.736	<0.001	***	1092.349	<0.001	***
Maternal treatment	3	3.933	0.269		3.054	0.383		6.191	0.103	
Descendants treatment	3	1.174	0.759		8.743	0.033	*	3.630	0.304	
Maternal × Descendants	9	14.435	0.097	.	10.604	0.304		14.136	0.118	

transformations: ^a^log; ^b^^0.5; ^c^^0.2. Linear Mixed-effects Models (LMMs) included Year, Maternal treatment, Descendants treatment, Generation and the two-way interaction between Maternal and Descendants treatment. Transformations applied to the response variable are indicated. Significance is shown as * 0.05 > *P* ≥ 0.01; ** 0.01 > *P* ≥ 0.001; *** *P <* 0.001, and . reflects marginal effects (0.1 > *P* ≥ 0.05). Sample size was 640. Response variables are number (*N*) of secondary roots, maximum rooting depth, root biomass, root weight ratio (RWR), relative root branching, and relative rooting depth.

## Results

### Effects of predictability treatments on root traits of ancestors

**Figure 2:**
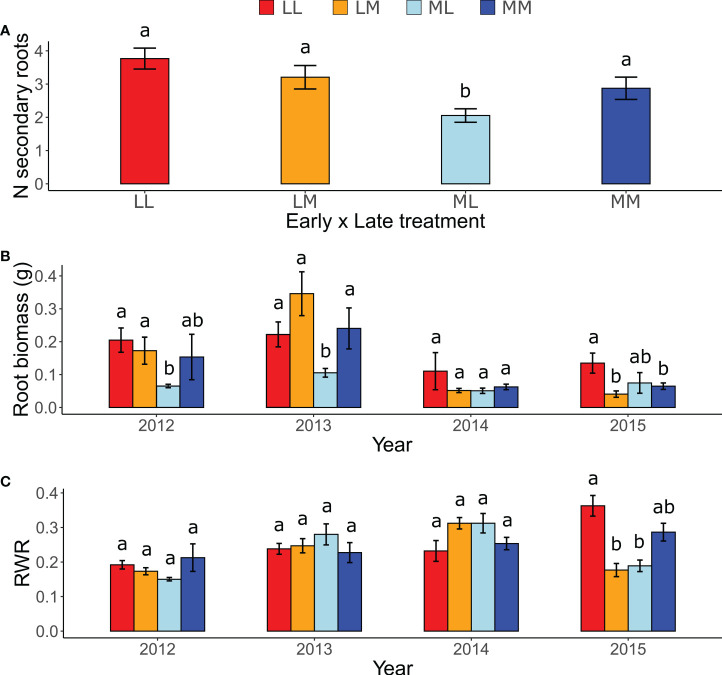
Effects of inter-seasonal predictability on the number of secondary roots, root biomass and root weight ratio (RWR) of ancestors. Interaction between: **(A)** Early treatment × Late treatment on the number (*N*) of secondary roots; **(B)** Early treatment, Late treatment, and Year on root biomass; and **(C)** Early treatment, Late treatment, and Year on RWR. Means ± SE are shown in the bar plots. Significant *post-hoc* contrasts among treatment combinations (and among treatments within the same year in B and C) are indicated with letters.

In the ancestral generation, which was sown in each year (2012-2015), the predictability treatment affected the response of all measured root traits except the maximum rooting depth. The number of secondary roots was significantly lower in ML than in the other treatment combinations (**Figure 2A**), as indicated by a significant early × late treatment interaction (**Table 1**). Moreover, although in 2013 plants in the more predictable late treatment (M) tended to have more secondary roots than in the less predictable late treatment (L; *P* = 0.06), late treatments did not differ in the other years (*P* ≥ 0.79), as indicated by a significant late treatment × year (**Table 1**). Root biomass was significantly higher in LL and LM than in ML in 2012 and 2013, and higher in LL than in ML and LM in 2015 (all *P* < 0.05; **Figure 2B**), as indicated by a significant three-way interaction between early treatment, late treatment and year (**Table 1**). However, no significant differences among treatments existed in 2014, and all other contrasts in 2012, 2013 and 2015 were not significant (*P* ≥ 0.17). For maximum rooting depth, there were no significant treatment effects (**Table 1**). Root weight ratio (RWR) was significantly higher in LL than in ML and LM in 2015 (**Figure 2C**), as indicated by a significant three-way interaction between early treatment, late treatment and year (**Table 1**). However, other treatments did not significantly differ in 2015, and all *post-hoc* contrasts in the other years were not significant (*P* ≥ 0.15). Relative root branching was significantly higher in the M than in the L early treatment (Early [M] = 0.449 ± 0.153 [Estimate ± SE]), and it was not affected by the late treatment (**Table 1**). Relative rooting depth was significantly higher in the L late treatment (Late [M] = -0.535 ± 0.171), and it was not affected by the early treatment (**Table 1**). So, overall, plants invested less biomass in roots and less in rooting depth but had increased root branching in the M treatment than in the L treatment, particularly if the treatment was imposed in spring.

### Selection acting on root traits

Selection gradients for the ancestral generation revealed significant non-linear relationships of total biomass (hereafter referred as ‘performance’) with maximum rooting depth (quadratic: Estimate ± SE = 0.158 ± 0.019, *P* < 0.001; linear: -0.817 ± 0.180, *P* < 0.001), RWR (quadratic: -0.087 ± 0.022, *P* < 0.001; linear: 0.670 ± 0.199, *P* < 0.001) and relative rooting depth (quadratic: 0.099 ± 0.016, *P* < 0.001, *P* < 0.001; linear: -1.784 ± 0.179, *P* < 0.001). Significant linear (but no quadratic) relationships also existed for the number of secondary roots (0.613 ± 0.039, *P* < 0.001) and relative root branching (-0.016 ± 0.001, *P* < 0.001). No significant differences in selection gradients existed among treatments (*i.e.* no significant interactions with treatment: χ_1_^2^ ≤ 3.21, *P* ≥ 0.08) in any of the root traits. Selection gradients for the number of secondary roots showed that performance, in terms of total biomass, increased with increasing number of secondary roots (**Figure S3A**). Performance decreased to 2.6 g (in the transformed data) and then increased again with increasing maximum rooting depth. The relationship between performance and RWR was convex and showed that performance was highest at a RWR of 3.9 (in the transformed data; **Figure S4A**). The relationship between performance and relative root branching was linear, and performance declined with increasing relative root branching (**Figure S4B**). The relationship between performance and relative rooting depth was concave and the higher the investment into rooting depth the smaller was the decline in performance (**Figure S4C**). Additionally, selection gradients for the different root traits did not significantly differ among ancestors and descendants (all: χ_1_^2^ ≤ 3.81, *P* ≥ 0.05). So, overall, root traits were generally under selection, and the predictability treatments did neither affect the strength nor the direction of selection.

### Transgenerational effect mediated by precipitation predictability

**Figure 3:**
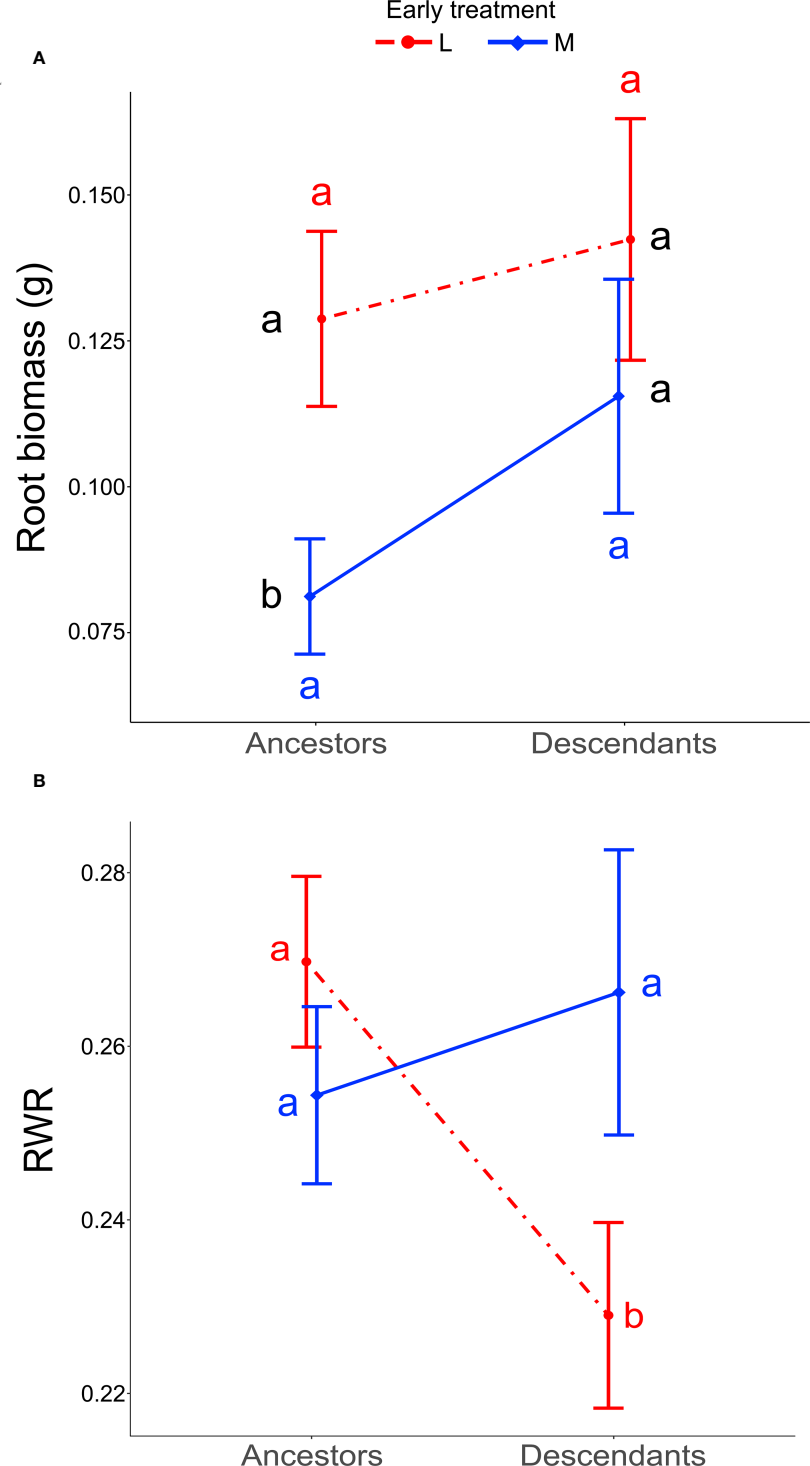
Differences between ancestors and descendants in root biomass and RWR. Shown are means ± SE of the two significant two-way interactions: Early treatment × Generation (ancestors *versus* descendants) on root biomass **(A)** and RWR **(B)**. Black letters represent *post-hoc* contrasts between both treatments within ancestors or within descendants. Red and blue letters represent *post-hoc* contrasts between ancestors and descendants in the less and in the more predictable treatment, respectively.

**Figure 4:**
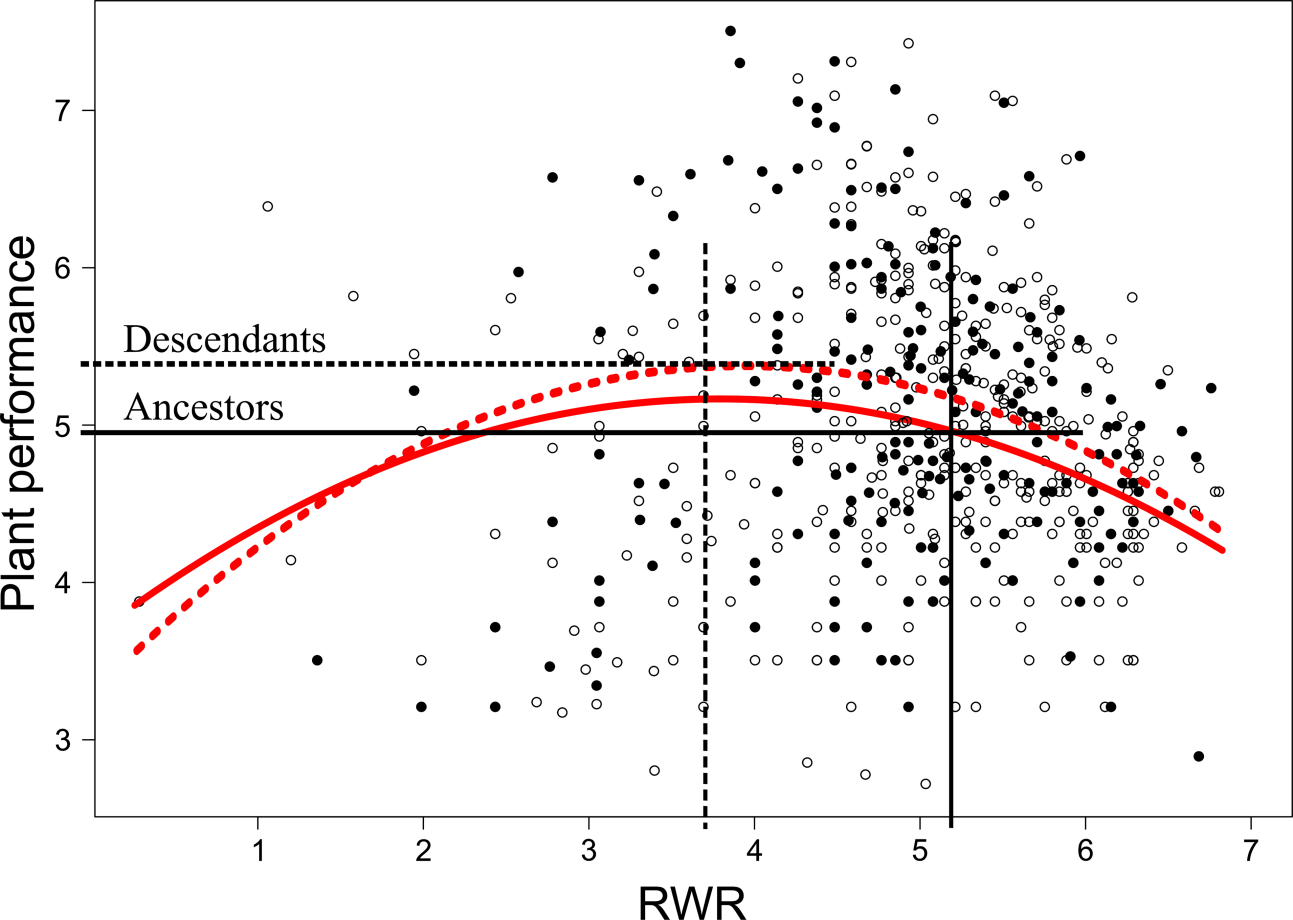
Non-linear selection gradients on root weight ratio (RWR) for ancestors and descendants in the less predictable early treatment. Solid and dashed red lines represent ancestors and descendants selection gradients, respectively. White and black dots represent data of ancestors and descendants, respectively. Average RWR of ancestors and descendants exposed to the less predictable early treatment (see **Figure 3B**) are indicated with vertical black lines and differences in plant performance between ancestors and descendants are indicated with horizontal black lines.

When testing for trait differences between ancestors and descendants subjected to high inter-seasonal predictability (only possible from 2013 to 2015), we found a significant two-way interaction between early treatment and generation (*i.e.*, ancestor vs. descendant) for root biomass (χ_1_^2^ = 5.10, *P* = 0.024) and for RWR (χ_1_^2^ = 4.86, *P* = 0.027). Root biomass of ancestors was significantly larger in the less compared to the more predictable early treatment (*post-hoc* contrast: *P* = 0.012), but no significant differences existed between early treatment levels in descendants in all three years (contrast: *P* = 0.4; **Figure 3A**). There were no significant differences between ancestors and descendants in number of secondary roots and maximum rooting depth (main effect and all interactions including generation and treatment: *P* ≥ 0.05). RWR of descendants was significantly lower than RWR of ancestors in the less predictable treatment (*P* = 0.022), whereas no differences existed between ancestors and descendants in the more predictable treatment (*P* = 0.22; **Figure 3B**). The significant difference in the less predictable early treatment led to a 13.42% increase in performance of descendants compared to ancestors (**Figure 4**). There were neither significant differences between ancestors and descendants in relative root branching and relative rooting depth nor for the interaction of generation with the late treatment in root biomass and RWR (main effect and all interactions including generation and treatment: *P* ≥ 0.1). So, overall, early predictability treatment induced changes between ancestors and descendants in root biomass and RWR but not in other root traits, which maintained or increased performance in descendants compared to in ancestors.

**Figure 5:**
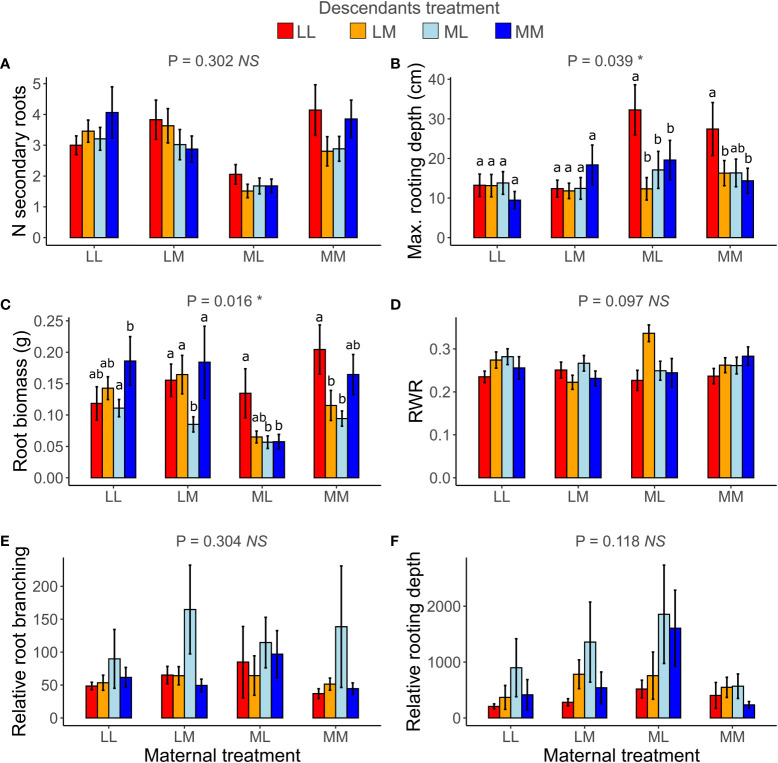
Transgenerational plasticity of roots in response to precipitation predictability treatments. Maternal treatment × Descendants treatment interaction in number (*N*) of secondary roots **(A)**, maximum (max.) rooting depth **(B)**, root biomass **(C)**, root weight ratio (RWR) **(D)**, relative root branching **(E)**, and relative rooting depth **(F)**. Means ± SE are shown in the bar plots. Significance of the Maternal treatment × Descendants treatment interaction is shown above each graph. Significant *post-hoc* contrasts among treatment combinations from the same maternal treatment are indicated with letters.

When testing for transgenerational plasticity to the different precipitation predictability treatments, we observed that the interaction between maternal treatment and descendants treatment was significant for maximum rooting depth and root biomass but not for the other root variables (**Table 2** and **Figure 5**). In both traits, there were no significant contrasts among descendant treatments within the same maternal treatment, but the results show that the effect of the descendants’ treatment depended on the maternal treatment. Descendants’ treatments significantly affected relative root branching (*i.e.*, independent of maternal treatment) and marginally affected the number of secondary roots and maximum rooting depth, but descendants’ treatments did not affect the other traits (**Table 2**). *Post-hoc* contrasts revealed that relative root branching was higher in ML than in the other descendant treatment combinations (*P* = 0.044; **Figure 5E**). Moreover, the lowest coefficients of variation (CV_m_) among treatments of descendants occurred in the maternal LL treatment in all measured root traits except for relative rooting depth, where the lowest CV_m_ occurred in the maternal MM treatment (**Table S1**). The greatest CV_m_ occurred in the maternal MM treatment for number of secondary roots and relative root branching, in the maternal ML treatment for root biomass and RWR, and in the maternal LM treatment for maximum rooting length and relative rooting depth (**Table S1**). So, overall, transgenerational plasticity existed in maximum rooting depth and root biomass, and the root response of descendants was not greater under the same (compared to different) maternal predictability treatment. Also, the coefficients of variation showed that generally the lowest plasticity is found in the maternal LL treatment. In addition, CVs of the descendants were greater than the CVs of the ancestors in all root traits except for number of secondary roots, suggesting greater plasticity in descendants than in ancestors.

## Discussion

Our multi-generation experiment demonstrates that precipitation predictability elicits intra- and trans-generational plasticity in root traits, and we observed differential root trait responses between ancestors and descendants of *Papaver rhoeas*. In ancestors, the magnitude of intra-generation plasticity most often depended on the year, and roots responded to lower predictability to increase water absorbance from shallow soil layers (*i.e.*, with greater number of secondary roots). Under high inter-generational predictability, ancestors and descendants exhibited differential root allocation strategies. In the less predictable early treatment, the relative investment into roots (Root weight ratio: RWR) was smaller in descendants compared to ancestors, reflecting a strategy that descendants used to increase their performance. These results are in line with studies suggesting that *P. rhoeas* can increase its average fitness and competitive ability during drought (e.g. Pérez-Ramos et al., 2019) and with a meta-analysis showing that transgenerational effects should be favoured in fast-growing short-lived organisms such as annual plants (Yin et al., 2019). The results also point to transgenerational plasticity, given that maternal predictability influenced the descendants’ plasticity in root traits. Moreover, transgenerational plasticity in root traits was lower when the maternal environment was less predictable, in line with theory and experiments with other systems (McNamara et al., 2016; Yin et al., 2019; Leung et al., 2020; Rescan et al., 2020). Our study thus provides experimental evidence for plastic adaptation in root traits in response to changes in precipitation predictability.

### Effects of predictability treatments on intra-generation root plasticity

The number of secondary roots, root biomass and root allocation strategies of ancestors were mediated by changes in precipitation predictability, revealing intra-generational plasticity. Treatment differences were absent in the maximum rooting depth and manifested only in a single year in the RWR. The number of secondary roots was lower in ML compared to the other treatment combinations, and LL exhibited, on average, the highest number (**Figure 2A**). These results may indicate a fast-acquisitive strategy to avoid drought by increasing water absorbance from shallow soil layers (Hickman, 1975; Koevoets et al., 2016; Weemstra et al., 2016). It also suggests that plants may adopt a less acquisitive strategy under more predictable water availability (Prieto et al., 2015; Weemstra et al., 2016). In line with our first hypothesis (H1), despite variation among years, root biomass was generally higher in LL conditions than in other treatment combinations (but not significantly higher in all years and between all treatments; see **Figure 2B**). Moreover, RWR was significantly higher in LL in 2015 (but no treatment differences existed in other years; **Figure 2C**), and relative rooting depth was significantly higher in the less predictable late treatment. These responses support the functional equilibrium hypothesis (Brouwer, 1983), which states that plants should allocate more to absorptive organs when resources are temporally scarce. Contrary, relative root branching was greater in the more predictable early treatment, which is in line with studies suggesting that more constant water availability favors increased root branching, *i.e.*, increased root density (e.g. Wu et al., 2016).

The observed plastic changes in roots with respect to predictability treatments are in agreement with the high plasticity found in root traits under environmental change (Hodge, 2004; Bardgett et al., 2014), but other experiments manipulating the amount of precipitation found little plasticity in root traits of herbaceous plants (e.g. Zhou et al., 2019). These differences in root plasticity between studies may be due to species-specific responses or due to differences in the applied treatment. For instance, previous studies have tested the effect of drought events on the root system, whereas we assessed root plasticity with regard to precipitation predictability while keeping the amount of obtained water constant among treatments.

### Selection acting on root traits

In line with the second hypothesis (H2), significant selection was acting on all measured root traits, showing that the measured root traits are functionally relevant (since they affect fitness; Violle et al., 2007). The number of secondary roots and relative root branching were under directional selection, whereas maximum rooting depth and relative rooting depth were under disruptive and RWR under stabilizing selection. Predictability treatments did not differently affect the strength or direction of selection acting on the measured traits. This implies that *P. rhoeas* may adopt the same strategies to increase performance under different degrees of intra- and inter-seasonal precipitation predictability.

Selection analyses also allow to understand which strategy optimizes performance, and they can reveal whether treatment-induced differences in trait averages led to an increase or decrease (*i.e.*, a constraint) in plant performance (Chevin, 2013). In ancestors, relative rooting depth was significantly higher in the less predictable late treatment while maximum rooting depth did not differ among treatments. The selection gradients showed that plant performance decreased with increasing relative rooting depth (**Figure S4C**) and that higher relative rooting depth under lower predictability only marginally decreased performance. *Papaver rhoeas* exposed to less predictable late treatment thus invested relatively more into rooting depth, but according to the selection gradients, this change in investment did not induce substantial performance benefits or losses. Moreover, ancestors of *P. rhoeas* reduced their relative root branching in the less predictable early treatment and the number of secondary roots was significantly bigger in LL and LM compared to ML (**Figure 2A**). According to the selection gradients, lower relative root branching and higher number of secondary roots resulted in higher performance. These findings indicate that, when conditions are less predictable, plants adjust their allocation to roots to maintain or increase their performance, revealing the functional role of roots to cope with different degrees of environmental predictability.

### Transgenerational effects mediated by precipitation predictability

Precipitation predictability elicited transgenerational effects in root biomass and RWR. In ancestors, root biomass was significantly higher in the less predictable early treatment compared to the more predictable early treatment, and in descendants no significant treatment differences existed (**Figure 3A**), pointing to transgenerational treatment-induced changes that minimized differences detected in ancestors. On average, root biomass was greater in descendants compared to ancestors under both less and more predictable early treatments. However, in line with our third hypothesis (H3), under more predictable precipitation, the average increase in root biomass from ancestors to descendants was greater than under less predictable conditions, suggesting that more predictable conditions allow for a stronger transgenerational response (McNamara et al., 2016; Dong et al., 2018). This suggests that transgenerational effects with respect to environmental predictability may balance effects on fitness over the course of generations (Donelson et al., 2018; Yin et al., 2019). Moreover, this indicates that high inter-generational predictability promotes gradual changes and may facilitate the evolution of transgenerational responses (Burgess and Marshall, 2014; McNamara et al., 2016; Yin et al., 2022). Under less predictable early treatment, descendants exhibited lower RWR than ancestors, whereas in more predictable early treatment no differences existed between ancestors and descendants (**Figure 3B**). The reduced RWR in descendants under less predictable conditions led to a 13.42% increase in performance (**Figure 4**), which points to an adaptive transgenerational effect (Herman and Sultan, 2011). This confirms that, in presence of natural environmental heterogeneity, intermediate environmental predictability, and in absence of extreme events, adaptive transgenerational effects may likely to occur (Burgess and Marshall, 2014; Uller et al., 2015; Yin et al., 2022).

The absence of treatment effects during the late growth phase and the absence of inter-seasonal predictability effects on the transgenerational response suggests that transgenerational effects principally occurred with respect to early growth conditions (when plants are most sensitive to environmental changes; Burton and Metcalfe, 2014). This is in line with results on phenological and fitness traits from previous experiments (March-Salas et al., 2019) and with a meta-analysis showing strongest transgenerational effects when environmental cues affect the juvenile phase (Yin et al., 2019). Moreover, there was no evidence for transgenerational changes in the investment in root branching and in root length, which suggests that plasticity in root traits might be conserved to successfully cope with differences in the predictability of precipitation.

Our experiment shows that precipitation predictability can drive transgenerational plasticity in root traits. All maternal predictability treatments promoted different root phenotypes among the descendants’ predictability treatments (**Figure 5**), and descendants generally showed a greater plasticity than ancestors (**Table S1**). In all measured root traits except for relative rooting depth (**Table S1**) the maternal LL treatment promoted a lower coefficient of variation in descendants compared to the other maternal treatments involving more predictable precipitation (*i.e.*, MM, ML, LM). This suggests that lower phenotypic plasticity evolves in less predictable environments, supporting previous findings in other systems (Leung et al., 2020; Rescan et al., 2020). This is also in line with simulations (McNamara et al., 2016), with experiments measuring reproductive traits of *Arabidopsis thaliana* (Yin et al., 2022) and with our own hypothesis (H4), suggesting that higher predictability generally favours transgenerational plasticity. In addition, descendants generally reduced their root response when less predictable conditions persisted over generations. This shows that plants can change their strategy if they grow under LL over consecutive generations. This is in line with the lower RWR in descendants compared to ancestors when conditions are less predictable. Our results also indicate that changes in plant strategies over generations may be favoured by the ancestors’ environment, in order to increase overall plant performance in the following generations (McIntyre and Strauss, 2014), and that adaptive transgenerational responses also occur under less predictable conditions (Donelson et al., 2018; Yin et al., 2019). However, a common-garden experiment will unravel whether root responses may be adaptive or merely plastic. Moreover, the direction and strength of transgenerational effect may vary across taxa, traits and developmental stages (Yin et al., 2019), or in presence of extreme events (Beier et al., 2012; Uller et al., 2015). Therefore, using model species and comparing subtle *versus* extreme changes in precipitation predictability may show more general and robust responses.

## Conclusion

Empirical evidence for evolutionary changes in response to differences in predictability is rare, especially in root traits. Our multi-generation experiment demonstrates that roots respond highly plastically to different degrees of precipitation predictability, that they are under strong selection pressure, and that transgenerational effects can enhance performance of descendants depending on the root trait and the predictability of environmental conditions. Lower predictability can enhance root responses and does not implicitly hinder a plant’s performance, since *P. rhoeas* altered its strategies involving root traits to maintain or even increase performance when reduced predictability persisted over generations. This points to adaptive transgenerational plasticity. However, a common-garden experiment growing offspring from all treatments under common conditions should be performed to confirm these findings. Overall, our findings show that even subtle changes in predictability elicit intra- and trans-generational plastic responses in root traits, highlighting the importance of environmental predictability as an evolutionary driver of transgenerational responses in plant populations.

## Data availability statement

The raw data supporting the conclusions of this article will be made available by the authors, without undue reservation.

## Author contributions

PF and MM-S designed the study with inputs from MK. MM-S implemented the study, measured the different traits and collected the samples with inputs from PF. MM-S analysed the data and wrote the manuscript, with PF, MK and JS contributing to revisions. All authors contributed to the article and approved the submitted version.

## Funding

Funding was provided by the Spanish Ministry of Economy and Competitiveness (CGL2012-32459, CGL2016-76918 to PSF) and the Swiss National Science Foundation (PPOOP3_128375, PP00P3_152929/1 to PSF). MM-S was supported by a PhD grant (BES-2013-062910) financed by the Spanish Ministry of Economy and Competitiveness. Publication costs were covered by Goethe University Frankfurt.

## Acknowledgments

We thank Guillermo Mercé, Blanca Santamaría, Diana Íñigo and Miguel Moreno for practical assistance, and María Urieta and Helena Clavero for technical assistance. We also thank Federico Fillat, Luis Villar, Paloma Mas and Guillem Masó for their scientific support.

## Conflict of interest

The authors declare that the research was conducted in the absence of any commercial or financial relationships that could be construed as a potential conflict of interest.

## Publisher’s note

All claims expressed in this article are solely those of the authors and do not necessarily represent those of their affiliated organizations, or those of the publisher, the editors and the reviewers. Any product that may be evaluated in this article, or claim that may be made by its manufacturer, is not guaranteed or endorsed by the publisher.

## Supplementary material

Supplementary Figure 1 Root diversity in example individuals of *Papaver rhoeas* from the experiment. From left to right: roots with decreasing numbers of secondary roots. The scale bar represents 50 mm.

Supplementary Figure 2 Temperature, potential evapotranspiration and precipitation at the study site. **(A)** Average daily temperature per month for each of the four experimental years. Colors and dot symbols correspond to the different experimental years and dotted lines to second order polynomial regressions. **(B)** Average potential evapotranspiration (PET) per month at the field site (Atlas Climático Digital de Aragón). The dotted line corresponds to a second order polynomial regression. **(C)** Difference between monthly precipitation (P) and potential evapotranspiration (PET) at the field site (red dots and red dotted line) and including the irrigated amount of water (yellow dots and yellow dotted line). Dotted lines correspond to second order polynomial regressions.

Supplementary Figure 3 Selection acting on root traits of ancestors. Model predictions of selection gradients are shown for number of secondary roots **(A)** and maximum rooting depth **(B)**. Since no significant interactions with treatments existed (*see* ‘Results’), only significant linear **(A)** and quadratic **(B)** predictions are shown.

Supplementary Figure 4 Selection acting on root traits indicating root allocation strategies of ancestors. Selection gradients are shown for root weight ratio (RWR) **(A)**, relative root branching **(B)**, and relative rooting depth **(C)**. Since no significant interactions with treatment existed (*see* ‘Results’), model predictions of significant quadratic **(A, C)** and linear **(B)** relationships are shown.

Supplementary Table 1 Means and coefficients of variation of measured root traits depending on maternal predictability treatments. The means of all root traits are shown for each of the descendant treatments depending maternal treatment, and also for each descendant treatment independent of the maternal treatment. The coefficient of variation (the ratio of the standard deviation to the mean, based on means, CV_m_) among treatments in descendants for each maternal treatment is also shown as well as the overall CV of ancestors (CV_a_) and the overall CV of descendants (CV_d_).

Supplementary Table 2 Sample size per treatment, year and generation. The sample size per treatment and year is presented for the ancestral plants, and the sample size per treatment and generation is presented for the descendants that were subjected to the same treatment for four generations (referred to as ‘descendants – pure lines’) and for the descendants from all treatment combinations over generations used for the analysis on transgenerational plasticity. The hypothesis (H) tested for each group of data is shown.
