# Supplementary material for: Precipitation predictability affects intra- and trans-generational plasticity and causes differential selection on root traits of Papaver rhoeas
Source: Front Plant Sci. 2022 Nov 14;13:998169. doi: 10.3389/fpls.2022.998169 (PMC9703072; doi:10.3389/fpls.2022.998169)

# SUPPORTING INFORMATION

SUPPLEMENTARY TEXT

***Soil and climatic conditions at the field site***

The field site shows Sub-Mediterranean climate, consisting of a temperate climate, with hot summers and moderate drought conditions throughout summer (Cfa, according to Beck et al. 2018; Loidi et al.2017; Fig. S2A). In April, when the experiment started and when seeds were sown, average daily temperatures were below 12°C and average daily maximal temperatures below 20°C (Fig. S2A). Thereafter, temperatures at the field site started to rise until July-August when average daily temperatures were above 20°C and average daily maximal temperatures were above 30°C (Fig. S2A). The period from late June to late August is relatively dry due to high evapotranspiration and the quick drying out of the soils (Figs. S2B and S2C).

The field site substrate consists of a permeable alluvial soil layer of approximately 50-60 cm, which exhibits good drainage, followed by a layer of coarse-grained compacted alluvial gravel. Soil moisture depends on precipitation, on the groundwater table depth, on evapotranspiration (e.g. Brouwer et al. 1985) and in this experiment, it also depends on irrigation. On the experimental site, the groundwater table depth is deeper than 1 meter, so none of the roots of the experimental plants reached the groundwater table. Moreover, precipitation falling at the field site exhibited no seasonality (*see* also March-Salas et al. 2019) but evapotranspiration varies with season and produces moderate drought conditions (Fig. S2B). Evapotranspiration depends on many factors, including solar radiation, wind and temperature (e.g. Brouwer et al. 1985). At the field site, the monthly potential evapotranspiration (PET) increases from 100 mm to 196 mm from April to July and thereafter it decreases again (Fig. S2B).

The difference between precipitation (*P*) and potential evapotranspiration (PET) is a measure of water stress (Gao and Giorgi, 2008). At the field site, during the growing season, the difference become more and more negative from April to July and thereafter it became less negative until September (Fig. S2C, red dots and curve). The difference became positive in October, after the growing season of *P. rhoeas* had finished. In other words, the difference between *P* plus the irrigated amount of water and PET is negative from June to August, and almost zero in May and September (Fig. S2C). This suggests that under natural conditions plants were exposed to water stress from April to September, which coincides with Gao and Giorgi’s (2008) findings that important water stress occurs in late spring and the summer months in this area, and with our observations that the natural grassland vegetation surrounding our experimental enclosures was drying out in early summer. In addition, as can be seen from Fig. S2C, *P* plus irrigation (yellow dots and yellow dotted line), led to a positive difference during seeding and sprouting, to a difference close to zero in May and in September, and to a negative difference from June to August. Finally, during the experiment, the soil of the plots was visibly dry, even during early treatment, *i.e.*, during periods when the difference between (*P* + irrigation) and PET was close to zero or positive.

**References (Supplementary text)**

Atlas Climático Digital de Aragón. <http://www.opengis.uab.es/wms/Aragon/index.htm>

Beck, H. E., Zimmermann, N. E., McVicar, T. R., Vergopolan, N., Berg, A., and Wood, E. F. (2018). Data Descriptor: Present and future Köppen-Geiger climate classification maps at 1-km resolution. Sci. Data 5(1), 180214

Brouwer, C. et al. (1985). Irrigation water management: training manual No. 1 – Introduction to Irrigation. FAO.

Gao, X., and Giorgi, F. (2008). Increased aridity in the Mediterranean region under greenhouse gas forcing estimated from high resolution simulations with a regional climate model. Glob. Plan. Change 62, 195–209.

Loidi, J. (2017). The Vegetation of the Iberian Peninsula. Volume 1. Springer.

March Salas, M., van Kleunen, M. and Fitze, P. S. (2019). Rapid and positive responses of plant to lower precipitation predictability. P. Roy. Soc. B 286, 20191486.

SUPPLEMENTARY TABLES

**Table S1.** Means and coefficients of variation of measured root traits depending on maternal predictability treatments. The means of all root traits are shown for each of the descendant treatments depending maternal treatment, and also for each descendant treatment independent of the maternal treatment. The coefficient of variation (the ratio of the standard deviation to the mean, based on means, CV_m_) among treatments in descendants for each maternal treatment is also shown as well as the overall CV of ancestors (CV_a_) and the overall CV of descendants (CV_d_).

| **Statistic** | **Maternal treatment** | **Descendants’ treatment** | **N secondary roots** | **Max. rooting depth** | **Root biomass** | **RWR** | **Relative root branching** | **Relative rooting depth** |
| --- | --- | --- | --- | --- | --- | --- | --- | --- |
| **Mean** | **LL** | **LL** | 3.038 | 13.430 | 0.121 | 0.235 | 49.526 | 239.829 |
|  |  | **LM** | 3.458 | 13.129 | 0.143 | 0.274 | 55.486 | 368.187 |
|  |  | **ML** | 3.308 | 13.803 | 0.111 | 0.282 | 87.143 | 859.189 |
|  |  | **MM** | 4.100 | 9.601 | 0.188 | 0.256 | 62.645 | 414.078 |
| **Mean** | **LM** | **LL** | 3.830 | 70.355 | 0.155 | 0.251 | 65.194 | 2453.348 |
|  |  | **LM** | 3.633 | 12.801 | 0.164 | 0.222 | 64.162 | 782.249 |
|  |  | **ML** | 3.020 | 13.429 | 0.085 | 0.267 | 164.717 | 1357.773 |
|  |  | **MM** | 2.875 | 18.364 | 0.184 | 0.231 | 49.433 | 540.635 |
| **Mean** | **ML** | **LL** | 2.056 | 32.252 | 0.135 | 0.227 | 82.829 | 518.069 |
|  |  | **LM** | 1.514 | 12.327 | 0.065 | 0.336 | 60.338 | 757.255 |
|  |  | **ML** | 1.679 | 17.101 | 0.057 | 0.249 | 115.531 | 1855.290 |
|  |  | **MM** | 1.680 | 19.596 | 0.058 | 0.244 | 97.865 | 1606.142 |
| **Mean** | **MM** | **LL** | 4.143 | 27.425 | 0.204 | 0.237 | 37.008 | 403.617 |
|  |  | **LM** | 2.805 | 16.292 | 0.115 | 0.262 | 51.410 | 548.286 |
|  |  | **ML** | 2.884 | 16.343 | 0.094 | 0.261 | 138.571 | 684.782 |
|  |  | **MM** | 3.853 | 14.355 | 0.165 | 0.293 | 44.550 | 234.677 |
| **Mean** | **All treatments** | **LL** | 3.401 | 40.178 | 0.150 | 0.240 | 56.515 | 463.263 |
|  |  | **LM** | 2.989 | 13.328 | 0.128 | 0.269 | 57.931 | 592.592 |
|  |  | **ML** | 2.827 | 14.579 | 0.091 | 0.267 | 127.127 | 1975.616 |
|  |  | **MM** | 3.186 | 15.508 | 0.155 | 0.253 | 60.178 | 637.057 |
| **CV_m_** | **LL** | **All treatments** | 0.130 | 0.156 | 0.244 | 0.080 | 0.259 | 0.573 |
|  | **LM** |  | 0.139 | 0.969 | 0.293 | 0.082 | 0.618 | 0.664 |
|  | **ML** |  | 0.132 | 0.419 | 0.479 | 0.186 | 0.262 | 0.546 |
|  | **MM** |  | 0.198 | 0.320 | 0.343 | 0.088 | 0.700 | 0.413 |
| **CV_a_** | **-** | **-** | 0.229 | 0.126 | 0.328 | 0.049 | 0.155 | 0.291 |
| **CV_d_** |  |  | 0.150 | 0.466 | 0.340 | 0.109 | 0.460 | 0.549 |

**Table S2.** Sample size per treatment, year and generation. The sample size per treatment and year is presented for the ancestral plants, and the sample size per treatment and generation is presented for the descendants that were subjected to the same treatment for four generations (referred to as ‘descendants – pure lines’) and for the descendants from all treatment combinations over generations used for the analysis on transgenerational plasticity. The hypothesis (H) tested for each group of data is shown.

| **Group** | **Treatment** | **Year** | **Generation** | **Sample size** |
| --- | --- | --- | --- | --- |
| **Ancestors (H1-H2)** | LL | 2012 | G0 | 153 |
|  | LM | 2012 | G0 | 216 |
|  | ML | 2012 | G0 | 12 |
|  | MM | 2012 | G0 | 48 |
|  | LL | 2013 | G0 | 177 |
|  | LM | 2013 | G0 | 119 |
|  | ML | 2013 | G0 | 87 |
|  | MM | 2013 | G0 | 82 |
|  | LL | 2014 | G0 | 42 |
|  | LM | 2014 | G0 | 61 |
|  | ML | 2014 | G0 | 42 |
|  | MM | 2014 | G0 | 76 |
|  | LL | 2015 | G0 | 80 |
|  | LM | 2015 | G0 | 56 |
|  | ML | 2015 | G0 | 61 |
|  | MM | 2015 | G0 | 67 |
| **Descendants - pure lines (H3)** | LL | 2013 | G1 | 94 |
|  | LM | 2013 | G1 | 126 |
|  | ML | 2013 | G1 | 18 |
|  | MM | 2013 | G1 | 83 |
|  | LL | 2014 | G2 | 25 |
|  | LM | 2014 | G2 | 28 |
|  | ML | 2014 | G2 | 20 |
|  | MM | 2014 | G2 | 25 |
|  | LL | 2015 | G3 | 34 |
|  | LM | 2015 | G3 | 15 |
|  | ML | 2015 | G3 | 16 |
|  | MM | 2015 | G3 | 18 |
| **Transgenerational plasticity (H4)** | LL | 2013 | G1 | 340 |
|  | LM | 2013 | G1 | 352 |
|  | ML | 2013 | G1 | 257 |
|  | MM | 2013 | G1 | 215 |
|  | LL | 2014 | G2 | 79 |
|  | LM | 2014 | G2 | 123 |
|  | ML | 2014 | G2 | 69 |
|  | MM | 2014 | G2 | 136 |
|  | LL | 2015 | G3 | 99 |
|  | LM | 2015 | G3 | 75 |
|  | ML | 2015 | G3 | 63 |
|  | MM | 2015 | G3 | 63 |

SUPPLEMENTARY FIGURES

**Figure S1.** Root diversity in example individuals of *Papaver rhoeas* from the experiment. From left to right: roots with decreasing numbers of secondary roots. The scale bar represents 50 mm.

**
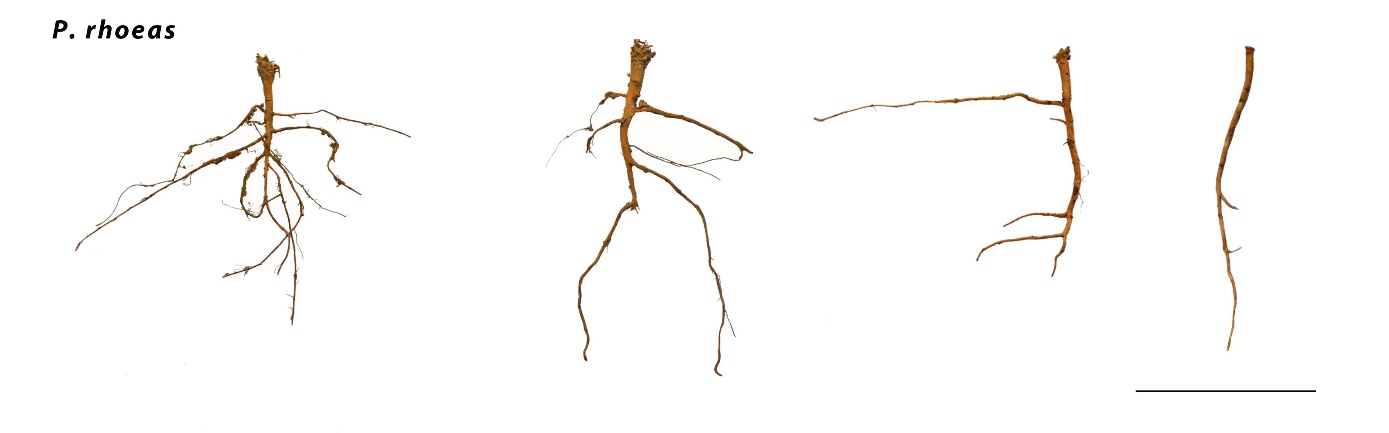
**

**Figure S2.** Temperature, potential evapotranspiration and precipitation at the study site. **(A)** Average daily temperature per month for each of the four experimental years. Colors and dot symbols correspond to the different experimental years and dotted lines to second order polynomial regressions. **(B)** Average potential evapotranspiration (PET) per month at the field site (Atlas Climático Digital de Aragón). The dotted line corresponds to a second order polynomial regression. **(C)** Difference between monthly precipitation (P) and potential evapotranspiration (PET) at the field site (red dots and red dotted line) and including the irrigated amount of water (yellow dots and yellow dotted line). Dotted lines correspond to second order polynomial regressions.

*
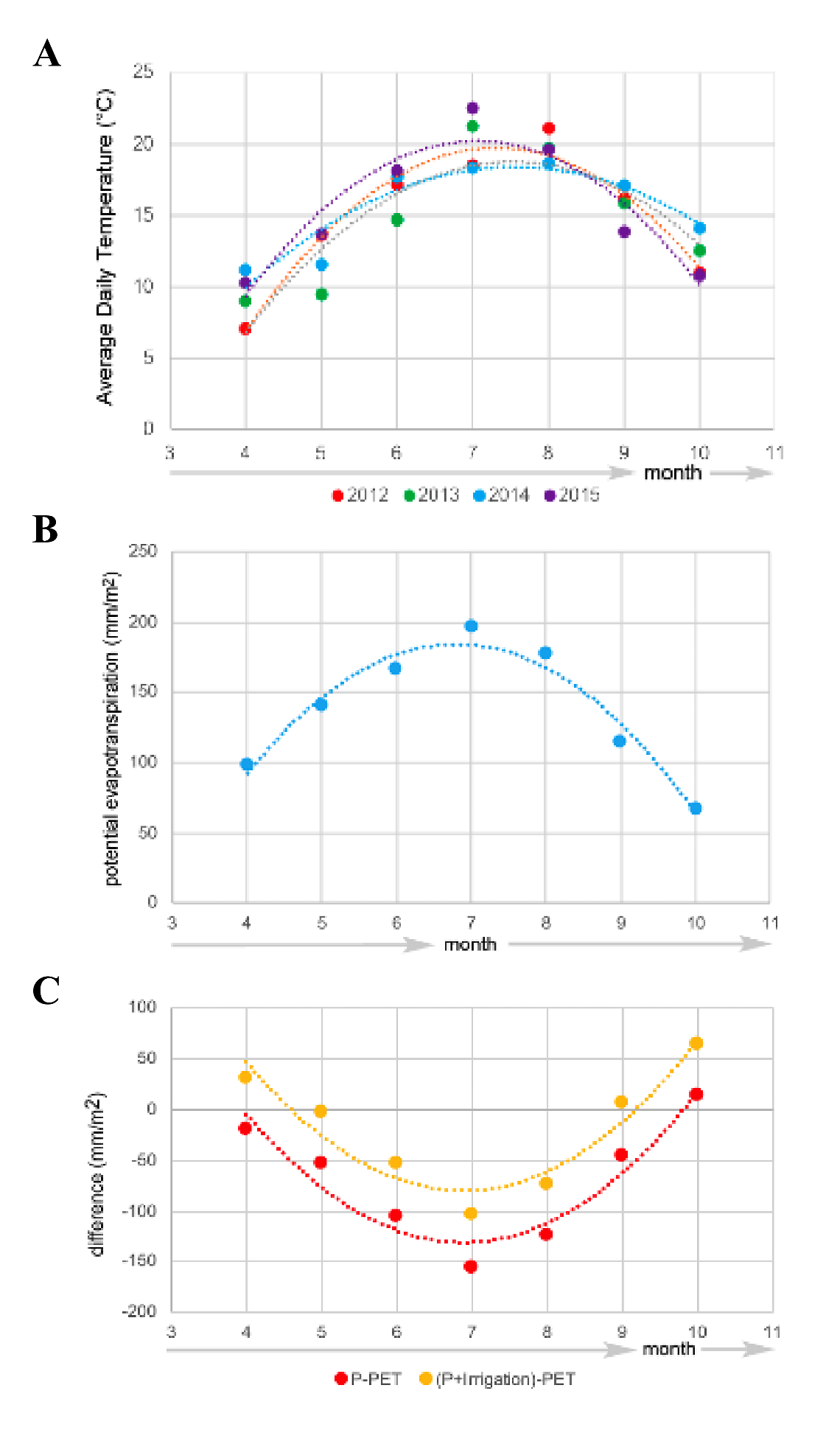
*

**Figure S3.** Selection acting on root traits of ancestors. Model predictions of selection gradients are shown for number of secondary roots **(A)** and maximum rooting depth **(B)**. Since no significant interactions with treatments existed (*see* ‘Results’), only significant linear (A) and quadratic (B) predictions are shown.

**
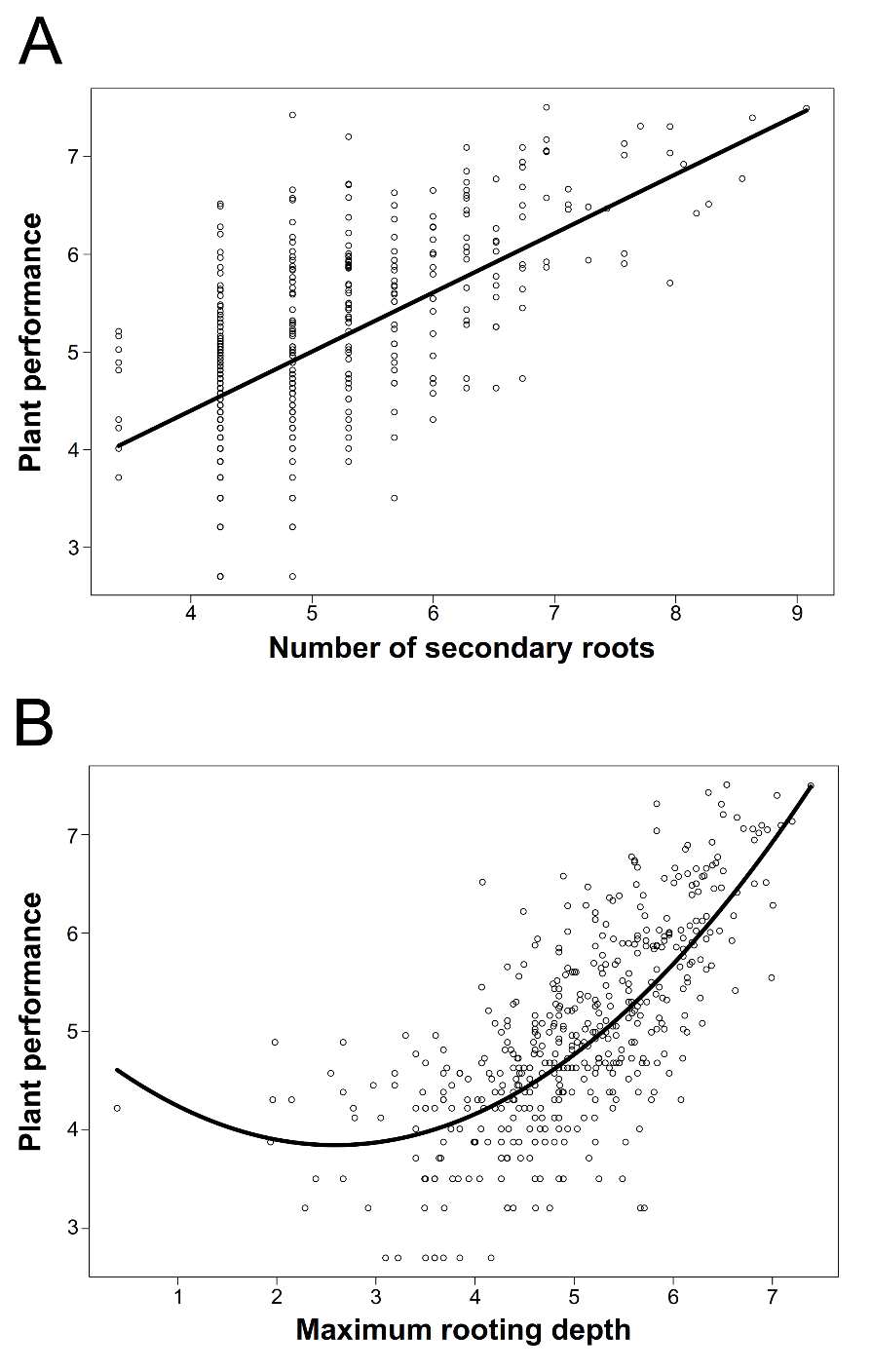
**

**Figure S4.** Selection acting on root traits indicating root allocation strategies of ancestors. Selection gradients are shown for root weight ratio (RWR) **(A)**, relative root branching **(B)**, and relative rooting depth **(C)**. Since no significant interactions with treatment existed (*see* ‘Results’), model predictions of significant quadratic (A, C) and linear (B) relationships are shown.


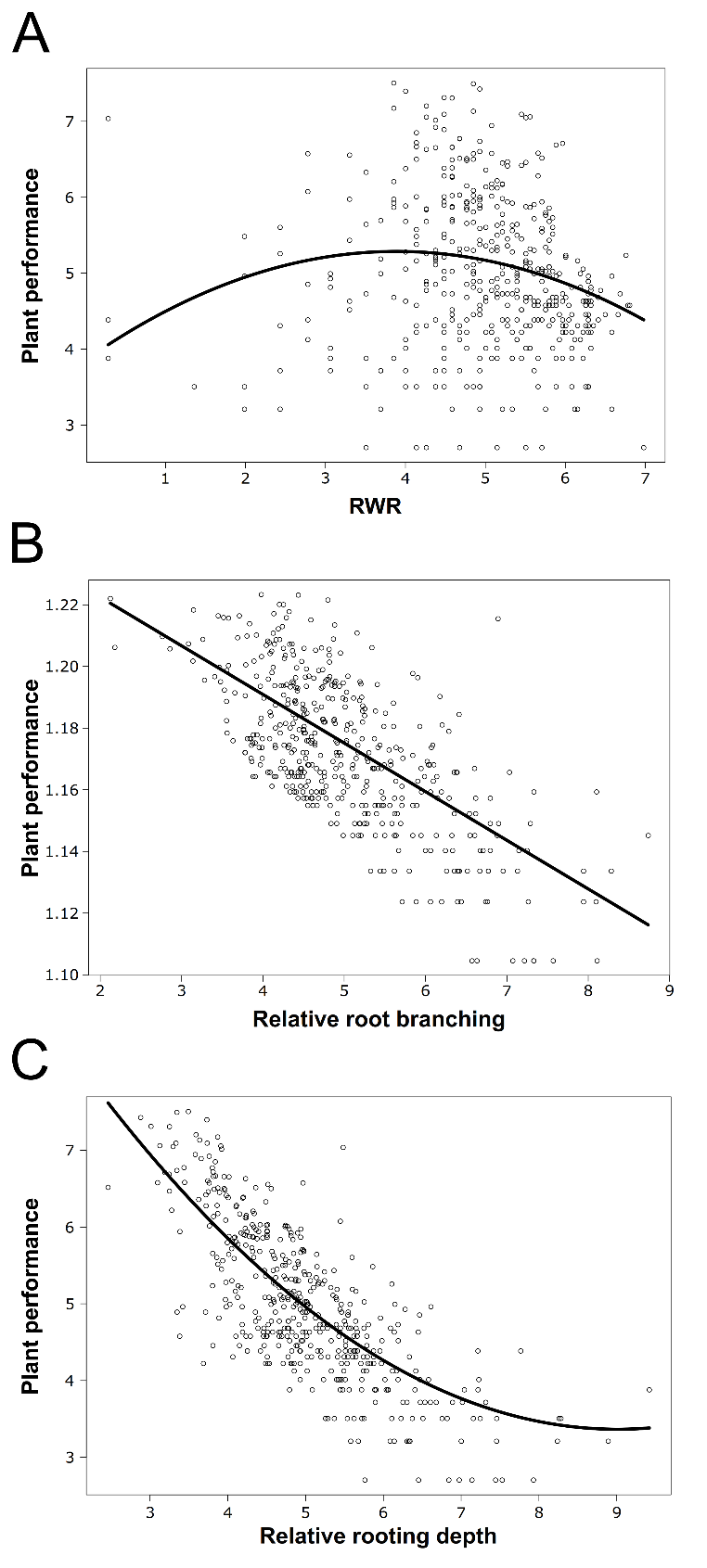

Supplement: Supplementary Figure 1 — Root diversity in example individuals of Papaver rhoeas from the experiment. From left to right: roots with decreasing numbers of secondary roots. The scale bar represents 50 mm. [file DataSheet_1.docx]
